# *QuickStats:* Percentage of Deaths from External Causes,* by Age Group^^†^^ — United States, 2017

**DOI:** 10.15585/mmwr.mm6832a7

**Published:** 2019-08-16

**Figure:**
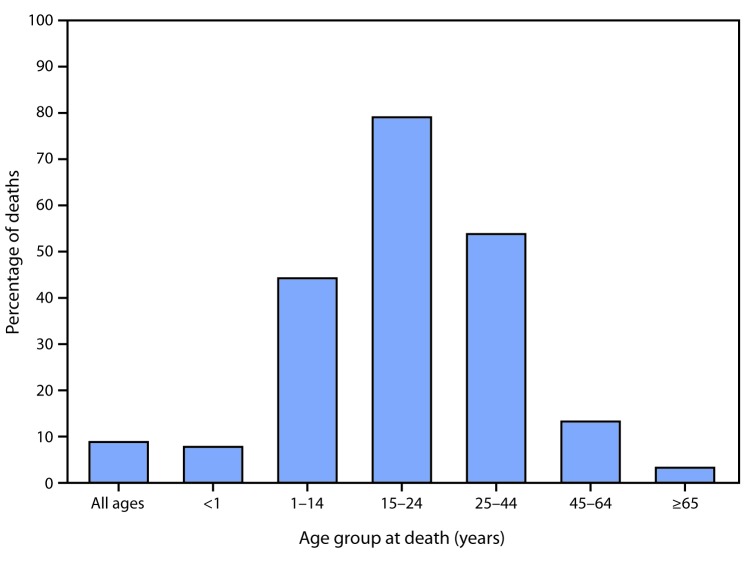
In 2017, 9% of all deaths were due to external causes. The percentage of deaths due to external causes was highest for those aged 15–24 years (79%) and lowest for those aged <1 year (8%) and aged >65 years (3%) at death. Among those aged 1–14 years, 44% of deaths were due to external causes, compared with 54% for those aged 25–44 years and 13% for those aged 45–65 years.

**Source:** National Vital Statistics System. Underlying cause of death data, 2017. https://wonder.cdc.gov/ucd-icd10.html.

For more information on this topic, CDC recommends the following link: https://www.cdc.gov/injury/.

